# ﻿Four new species and a new record of *Synagelides* Strand, 1906 (Araneae, Salticidae) from China

**DOI:** 10.3897/zookeys.1245.154908

**Published:** 2025-07-15

**Authors:** Bin-Lu Liu, Xiao-Qi Ning, Shi-Hui Huang, Yan-Bin Yao, Chang-Yong Lin, Jun Yang, Ke-Ke Liu

**Affiliations:** 1 Key Laboratory of Jiangxi Province for Biological Invasion and Biosecurity, Jinggangshan University, Ji’an, 343009, Jiangxi, China Jinggangshan University Ji’an China; 2 Jinshan College of Fujian Agriculture and Forestry University, Fuzhou 350007, Fujian, China Jinshan College of Fujian Agriculture and Forestry University Fuzhou China; 3 Tongboshan National Natrue Reserve, Shangrao, 334609, Jiangxi, China Tongboshan National Natrue Reserve Shangrao China; 4 Wuyuan Forest Birds National Nature Reserve, Shangrao, 333200, Jiangxi, China Wuyuan Forest Birds National Nature Reserve Shangrao China

**Keywords:** Ant-like, Jiangxi Province, jumping spider, taxonomy, Tibet Autonomous Region

## Abstract

Four new species of the jumping spider genus *Synagelides* Strand, 1906 are described from China: *S.dajueshan* K. K. Liu, **sp. nov.** (♂, ♀; Jiangxi Province), *S.guangfeng* K. K. Liu, **sp. nov.** (♂, ♀; Jiangxi Province), *S.kangding* K. K. Liu, **sp. nov.** (♂, ♀; Sichuan Province), and *S.wuyuan* K. K. Liu, **sp. nov.** (♂, ♀; Jiangxi Province). Furthermore, *S.himalaicus* Bohdanowicz, 1987, **stat. res.** (♂, ♀; Tibet Autonomous Region) is reinstated as a valid species and recorded from China for the first time, with *S.jiricus* Bohdanowicz, 1987, **syn. nov.** designated as its junior synonym. All species are comprehensively illustrated with diagnostic photographs and SEM images, and a distribution map is also provided. The geographic distribution of this genus in China and their adaptations are discussed.

## ﻿Introduction

*Synagelides* Strand, 1906 is amongst the most diverse genera of jumping spiders (Salticidae). To date, 85 species are known worldwide, many of which are ant-mimics; 53 species are recorded from China (The Biodiversity Committee of Chinese Academy of Sciences 2024; WSC 2025). In China, most species were described from the southern provinces, and only three species were recorded from the northern provinces: Anhui (1 species), Guangxi (7 species), Guizhou (10 species), Hainan (1 species), Hubei (7 species), Hunan (4 species), Sichuan (1 species), Yunnan (15 species), Zhejiang (1 species), Jilin (2 species), Hebei (1 species), Jiangsu (1 species), Chongqing (2 species), Tibet (3 species), Taiwan (3 species), and Jiangxi (10 species). The fragmented distribution patterns of these species strongly suggest the existence of undocumented diversity.

During examinations of spider specimens collected from the Tibet Autonomous Region and Sichuan and Jiangxi provinces of China, four new species of *Synagelides* were found, which are described herein. Additionally, *S.himalaicus* Bohdanowicz, 1987 stat. res. is reinstated as a valid species and newly recorded from China, with *S.jiricus* Bohdanowicz, 1987, syn. nov. established as its junior synonym.

## ﻿Materials and methods

Specimens were examined using a Zeiss Stereo Discovery V12 stereomicroscope equipped with a Zeiss AxioCam HRc. Male palps and female epigynes were dissected and studied in 80–85% ethanol. Vulvae were cleaned using pancreatin. All specimens were photographed with an Olympus CX43 compound microscope fitted with a KUY NICE CCD camera (Beijing Tiannuoxiang Scientific Instrument Co., Ltd., China).

For scanning electron microscopy (SEM), specimens were either air-dried and gold-coated using a small ion-sputtering apparatus (ETD-2000; Beijing Yilibotong Technology Development Co., Ltd., China) or examined uncoated, and imaged using a Zeiss EVO LS15 scanning electron microscope (Carl Zeiss AG, Germany). Images were processed using the ImagineView software package and the Smart SEM User Interface.

All measurements were taken with a stereomicroscope using the AxioVision SE64 Rel. 4.8.3 software and are presented in millimeters. Leg measurements are given as total length, with individual segments listed in the following order: femur, patella, tibia, metatarsus, and tarsus. All specimens are deposited in the Animal Specimen Museum, College of Life Science, Jinggangshan University (ASM-JGSU).

The terminology for male and female copulatory organs follows Liu et al. (2022), Omelko and Fomichev (2021), and Li et al. (2021). The abbreviations used in the figures are as follows:

Eyes

**ALE** anterior lateral eye;

**AME** anterior median eye;

**PLE** posterior lateral eye;

**PME** posterior median eye;

**MOA** median ocular area.

Legs

**ti** tibia;

**pv** proventral;

**rv** retroventral;

**met** metatarsus.

Male palp

**DTA** dorso-prolateral tibial apophysis;

**DCA** dorsal cymbial apophysis;

**Em** embolus;

**PCA** postero-prolateral cymbial apophysis;

**TS** tibial spine;

**RCA** postero-retrolateral cymbial apophysis;

**RTA** retrolateral tibial apophysis;

**SS** scale-like serrations;

**TA** terminal apophysis;

**VFA** ventral femoral apophysis.

Epigyne

**AR** atrial rim;

**At** atrium;

**CD** copulatory duct;

**CO** copulatory opening;

**EH** epigynal hood;

**FD** fertilization duct;

**GA** glandular appendage;

**MS** median septum;

**Spe** spermatheca.

## ﻿Taxonomy

### ﻿Family Salticidae Blackwall, 1841

**Tribe Agoriini Simon, 1901 (sensu Maddison 2015)**

**Genus *Synagelides* Strand, 1906**

#### ﻿ Synagelides dajueshan

Taxon classificationAnimaliaAraneaeSalticidae

K. K. Liu sp. nov.

B748DBBF-9CA1-51CF-A76A-CA9E24CEEA6A

https://zoobank.org/79E38096-7C85-4C0A-B0E7-4EB17ACCB623

Figs 1 , 2 , 3

##### Material examined.

***Holotype*** • 1 ♂ (Sal-414, ASM-JGSU), 27°41'47.65"N, 117°10'28.80"E, 722 m a.s.l., Dajue Mountain, Zixi County, Fuzhou City, Jiangxi Province, China, 4 October 2024, K. Liu, X. Chen, Y. Shi, X. Lyu & Z. Wang leg. ***Paratypes*** • 1 ♂ 2 ♀ (Sal-414, ASM-JGSU), same data as holotype.

##### Diagnosis.

**Figure 1.**
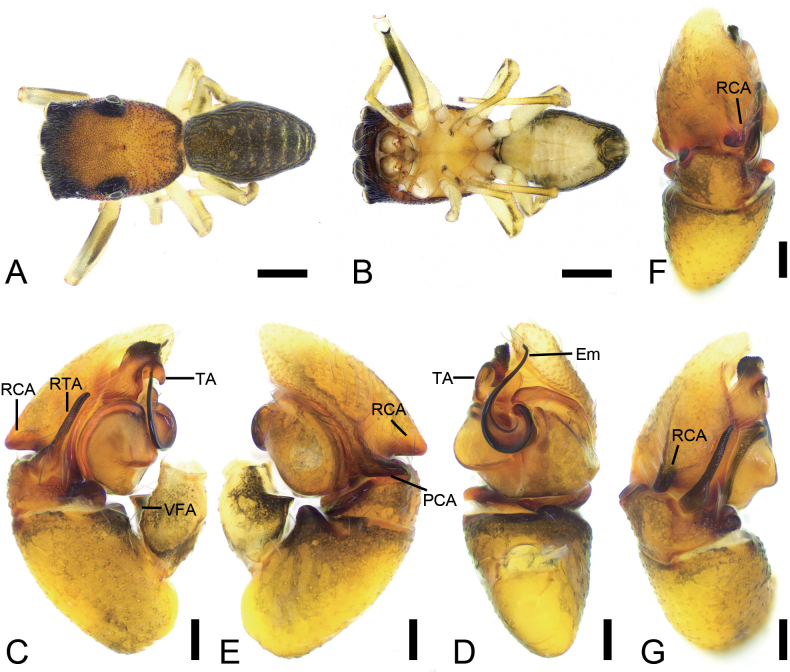
*Synagelidesdajueshan* sp. nov., male holotype. **A.** Habitus, dorsal view; **B.** Same, ventral view; **C.** Right palp, retrolateral view; **D.** Same, ventral view; **E.** Same, prolateral view; **F.** Same, dorsal view; **G.** Same, postero-retrolateral view, slightly dorsal. Abbreviations: Em – embolus, PCA – postero-prolateral cymbial apophysis, RCA – postero-retrolateral cymbial apophysis, RTA – retrolateral tibial apophysis, TA – terminal apophysis, VFA – ventral femoral apophysis. Scale bars: 0.5 mm (**A, B**); 0.1 mm (**C–G**).

**Figure 2.**
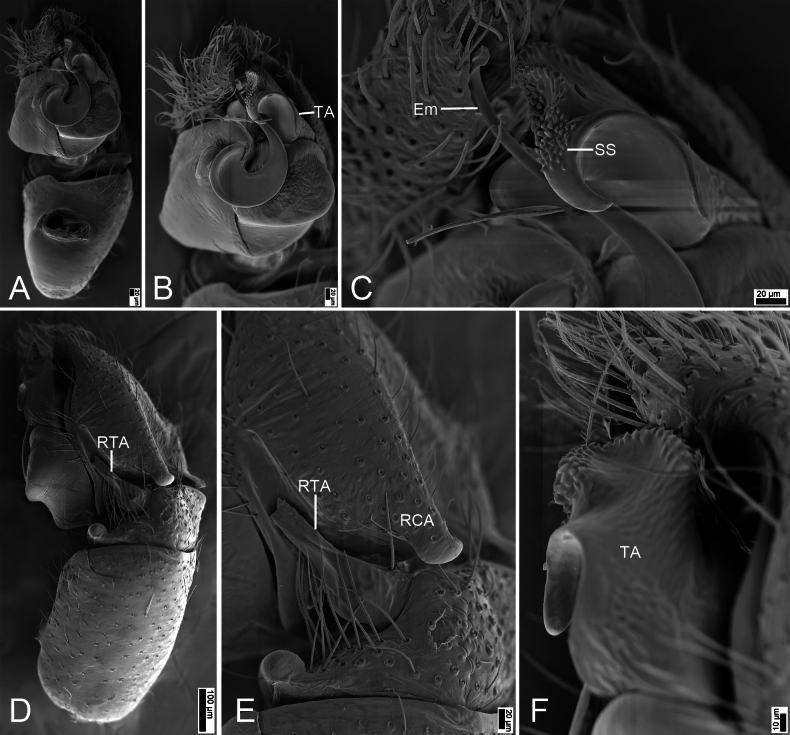
*Synagelidesdajueshan* sp. nov., SEM images of the holotype male, left palp. **A.** Palp, ventral view; **B.** Same, detail of terminal apophysis and embolus; **C.** Same, detail of terminal apophysis and embolus; **D.** Same, retrolateral view; **E.** Same, detail of retrolateral tibial apophysis and postero-retrolateral cymbial apophysis; **F.** Same, detail of terminal apophysis. Abbreviations: Em – embolus, RCA – postero-retrolateral cymbial apophysis, RTA – retrolateral tibial apophysis, SS – scale-like serrations, TA – terminal apophysis. Scale bars: 20 μm (**A–C, E**); 100 μm (**D**); 10 μm (**F**).

**Figure 3.**
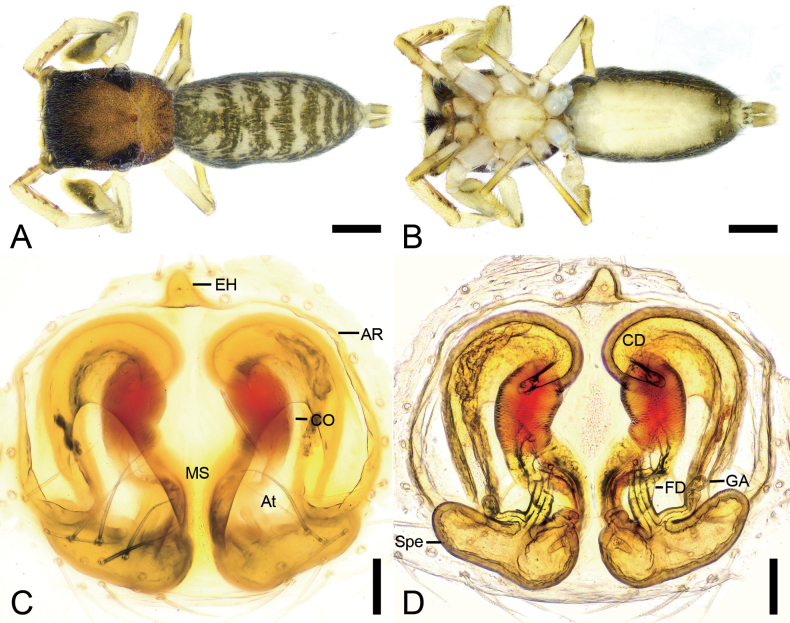
*Synagelidesdajueshan* sp. nov., female paratype. **A.** Habitus, dorsal view; **B.** Same, ventral view; **C.** Epigyne, ventral view; **D.** Same, dorsal view. Abbreviations: AR – atrial rim, At – atrium, CD – copulatory duct, CO – copulatory opening, EH – epigynal hood, FD – fertilization duct, GA – glandular appendage, MS – median septum, Spe – spermatheca. Scale bars: 0.5 mm (**A, B**); 0.2 mm (**C, D**).

The male of this species is similar to that of *Synagelidesjinding* Liu, 2022 in having flattened discoid embolus and hook-shaped terminal apophysis, but can be distinguished by the embolus tip curving retrolaterally (vs straight); the slender retrolateral tibial apophysis with broad base, gradually tapering distally and slightly curved apically (vs broader at both base and distal end but narrower medially); and the inconspicuous dorso-prolateral tibial apophysis (vs prominent) (Figs 1C–G, 2A–F cf. Liu et al. 2022: 66, figs 4A–G, 5A–G). The female of this species is similar to that of *S.jinggangshanensis* Liu, Chen, Xu & Peng, 2017 in having the apple-like epigynal plate and the S-shaped anterior part and the slightly swollen medial part of copulatory duct, but can be easily distinguished by the short finger-shaped epigynal hood (vs distinctly long finger-shaped), the inverted triangular median septum (vs T-shaped); the copulatory openings located sub-anterolateral part of epigynal plate (vs along the midline); the slight swollen posterior part of copulatory duct (vs thin); and the very long glandular appendages nearly as long as 3/4 length of spermathecae (vs short, nearly as long as 1/2 length of spermathecae) (Fig. 3C, D cf. Liu et al. 2017: 292, figs 1A–D, 2A, B).

##### Description.

**Male** (holotype, Sal-414). Habitus as in Fig. 1A, B. Total length 2.84. Carapace 1.44 long, 1.07 wide. Eye sizes and interdistances: AME 0.28, ALE 0.19, PME 0.05, PLE 0.19, AME–AME 0.05, AME–ALE 0.08, PME–PME 0.87, PME–PLE 0.29, AME–PME 0.46, AME–PLE 0.77, ALE–ALE 0.72, PLE–PLE 0.94, ALE–PLE 0.63. MOA: 0.61 long; 0.64 anterior width, 0.95 posterior width. Chelicerae (Fig. 1B) with two promarginal teeth and one large triangular retromarginal tooth. Sternum (Fig. 1B) shield-shaped, longer than wide, posterior end triangular, smooth. Leg measurements: I 3.19 (1.01, 0.72, 0.81, 0.38, 0.27); II 2.03 (0.61, 0.27, 0.53, 0.34, 0.28); III 2.15 (0.64, 0.39, 0.35, 0.50, 0.27); IV 2.98 (0.92, 0.38, 0.62, 0.66, 0.40). Femur width: I 0.25; II 0.19; III 0.22; IV 0.23. Leg spination (Fig. 1A, B): I ti pv1-2-1, rv1-2-1; met pv1-0-1, rv1-0-1. Pedicel 0.03. Abdomen 1.37 long, 0.85 wide.

***Coloration*** (Fig. 1A, B). Carapace reddish-brown to dark brown dorsally, densely covered with reddish-brown punctate spots. Eye bases surrounded by black pigmentation. Fovea distinct, encircled by several radial rows of punctate striae. Chelicerae yellowish-brown. Endites yellowish-brown, wider than long. Sternum yellow, mottled, with densely dark brown mottled spots. Legs yellowish-brown to dark brown; leg I yellow, with dark brown longitudinal bands pro- and retrolaterally. Abdomen dark brown, mottled, with five chevron-shaped yellowish stripes in posterior part; venter with a pair of yellow brown stripes. Spinnerets dark yellow-brown, mottled. Spinnerets dark yellow-brown, mottled.

***Palp*** (Figs 1C–G, 2A–F). Femur with a tooth-like ventral apophysis. Patella swollen, with a length–width ratio of ca 1.73. Retrolateral tibial apophysis elongate, broad basally and progressively narrowing toward the slightly curved distal end. Postero-prolateral cymbial apophysis digitiform, with sharply pointed apex. Postero-retrolateral cymbial apophysis short and finger-shaped. Tegulum broad, with a clear mastoid apophysis in retrolateral view. Terminal apophysis arising from antero-retrolateral part of tegulum, strongly sclerotized, hook-shaped, C-shaped in retrolateral view, with abundant little scale-like serrations on apical surface. Embolus with a flattened discoid base, gradually tapering and retrolaterally curved distally.

**Female** (paratype, Sal-414). Habitus as in Fig. 3A, B. As in male, except as noted. Total length 3.42. Carapace 1.47 long, 1.10 wide. Eye sizes and interdistances: AME 0.30, ALE 0.18, PME 0.04, PLE 0.18, AME–AME 0.08, AME–ALE 0.08, PME–PME 0.88, PME–PLE 0.29, AME–PME 0.46, AME–PLE 0.78, ALE–ALE 0.71, PLE–PLE 0.98, ALE–PLE 0.65. MOA: 0.63 long; 0.71 anterior width, 0.93 posterior width. Sternum (Fig. 3B) shield-shaped, longer than wide, anterior margin slightly straight. Leg measurements: I 2.85 (0.90, 0.56, 0.77, 0.35, 0.27); II 2.16 (0.68, 0.36, 0.42, 0.43, 0.27); III (0.72, 0.33, 0.42, other segments broken); IV 3.08 (0.90, 0.38, 0.72, 0.74, 0.34). Femur width: I 0.29; II 0.20; III 0.19; IV 0.25. Leg spination (Fig. 3A, B): I ti pv1-2-1, rv1-2-1; met pv1-0-1, rv1-0-1. Pedicel 0.04. Abdomen 2.00 long, 1.01 wide.

***Coloration*** (Fig. 3A, B). Paler than male. Abdomen dorsally with several white longitudinal stripes; venter smooth, pale yellow.

***Epigyne*** (Fig. 3C, D). Epigynal plate apple-shaped, with relatively large atrium. Atrial rim ear-like, slightly sclerotized. Median septum bottle-neck shaped. Epigynal hood short finger-shaped, arising from anteromedial atrial rim. Copulatory openings distinct, located anterolateral part of medial septum, connecting to S-shaped copulatory ducts. Copulatory ducts very long, anterior part extending anteriorly, medial part slightly swollen, posterior part slightly swollen, slightly curved, convergent. Glandular appendages very long, longer than 3/4 length of spermathecae, directed laterally. Spermathecae boat-shaped, slightly separated, slightly shorter than 1/2 width of vulva. Fertilization ducts relatively long, nearly as long as 1/3 length of spermathecae, obliquely upward.

##### Distribution.

Known only from the type locality in Jiangxi Province, China (Fig. 16).

##### Etymology.

The name is taken from the type locality, noun in apposition.

#### ﻿ Synagelides guangfeng

Taxon classificationAnimaliaAraneaeSalticidae

K. K. Liu sp. nov.

56EF8C8E-963C-5B0B-BEC4-75A3044E0A53

https://zoobank.org/00C44BDE-22A1-4739-BDA8-C2437F9AE6C8

Figs 4 , 5 , 6

##### Material examined.

***Holotype*** • 1 ♂ (Sal-409, ASM-JGSU), 28°09'52.62"N, 118°19'31.66"E, 321 m a.s.l., Lingshang, Zhangduntou Station, Tongboshan National Nature Reserve, Tongboshan Town, Guangfeng District, Shangrao City, Jiangxi Province, China, 1 December 2024, K. Liu, C. Lin, Y. Shi, X. Lyu & Z. Wang leg. ***Paratypes*** • 2 ♀ (Sal-401, ASM-JGSU), 28°09'46.53"N, 118°17'45.81"E, 603 m a.s.l., Baihankengjiao, 11 July 2023, K. Liu, Z. Jiang & C. Li leg., other data same as previous.

##### Diagnosis.

**Figure 4.**
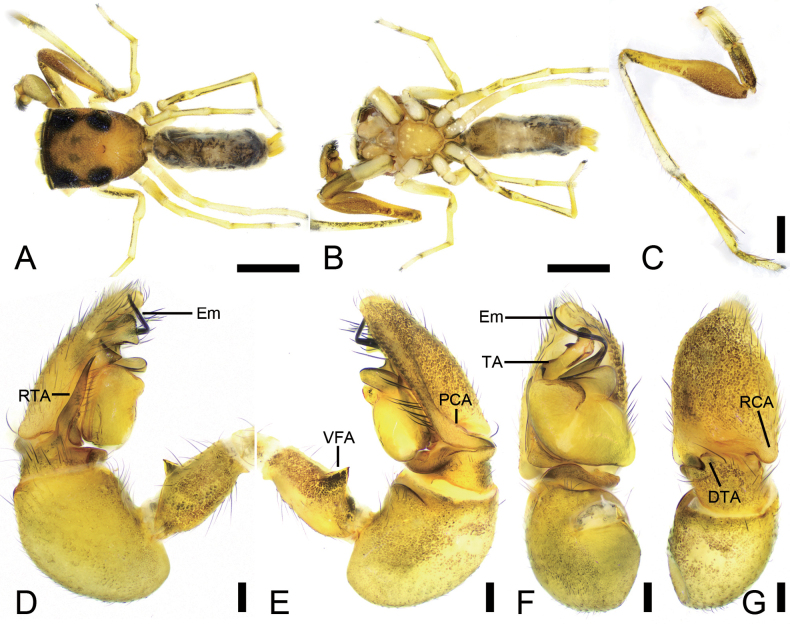
*Synagelidesguangfeng* sp. nov., male holotype. **A.** Habitus, dorsal view; **B.** Same, ventral view; **C.** Right leg I, retrolateral view; **D.** Right palp, retrolateral view; **E.** Same, prolateral view; **F.** Same, ventral view; **G.** Same, dorsal view. Abbreviations: DTA – dorso-prolateral tibial apophysis, Em – embolus, PCA – postero-prolateral cymbial apophysis, RCA – postero-retrolateral cymbial apophysis, RTA – retrolateral tibial apophysis, TA – terminal apophysis, VFA – ventral femoral apophysis. Scale bars: 1 mm (**A, B**); 0.1 mm (**C–G**).

**Figure 5.**
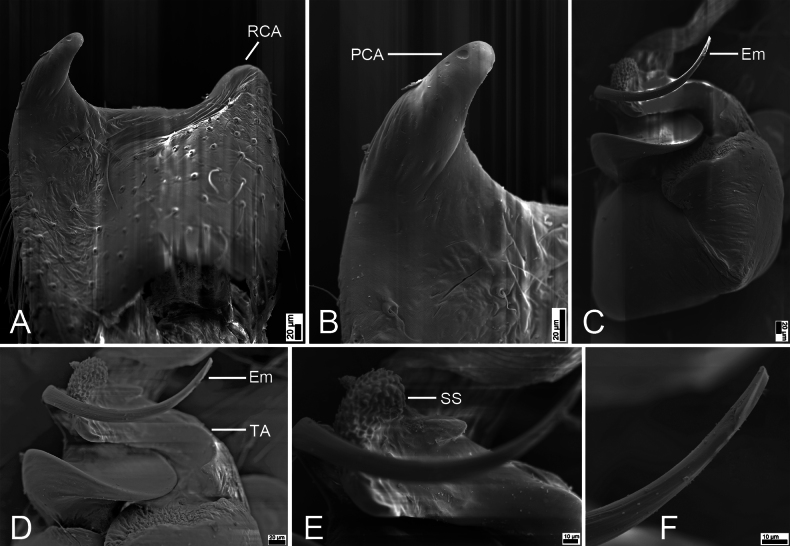
*Synagelidesguangfeng* sp. nov., SEM images of the holotype male, left palp. **A.** Cymbium, detail of postero-prolateral cymbial apophysis and postero-retrolateral cymbial apophysis; **B.** Same, detail of postero-prolateral cymbial apophysis; **C.** Palp, detail of terminal apophysis and embolus; **D.** Same, detail of terminal apophysis and embolus; **E.** Same, detail of terminal apophysis; **F.** Same, detail of embolus. Abbreviations: Em – embolus, PCA – postero-prolateral cymbial apophysis, RCA – postero-retrolateral cymbial apophysis, SS – scale-like serrations, TA – terminal apophysis. Scale bars: 20 μm (**A–D**); 10 μm (**E, F**).

**Figure 6.**
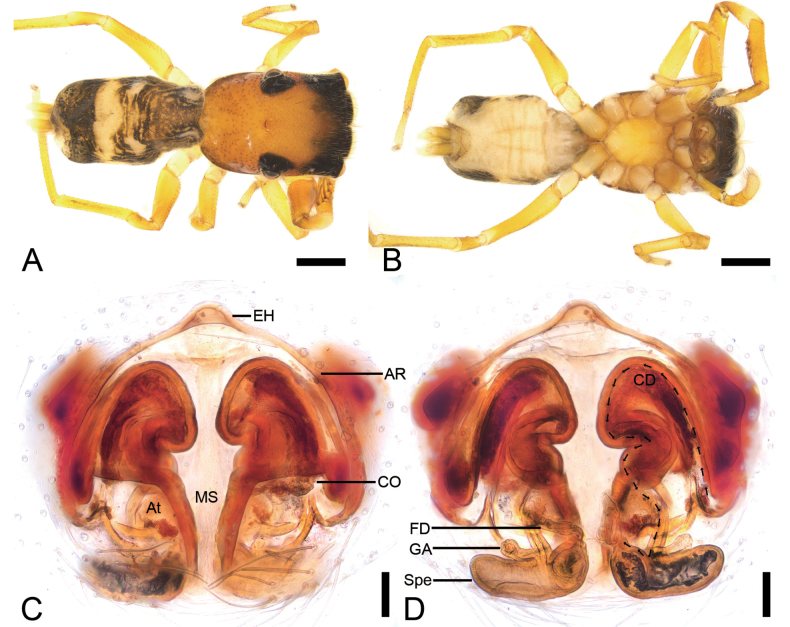
*Synagelidesguangfeng* sp. nov., female paratype. **A.** Habitus, dorsal view; **B.** Same, ventral view; **C.** Epigyne, ventral view; **D.** Same, dorsal view. Abbreviations: AR – atrial rim, At – atrium, CD – copulatory duct, CO – copulatory opening, EH – epigynal hood, FD – fertilization duct, GA – glandular appendage, MS – median septum, Spe – spermatheca. Scale bars: 0.5 mm (**A, B**); 0.1 mm (**C, D**).

The male of this species is similar to that of *Synagelideslongus* Song & Chai, 1992 in having anticlockwise extending and S-shaped embolus, but can be distinguished by the yellowish-brown body coloration (vs reddish-brown); the coxa and trochanter distinctly protruding (vs normal); the more robust and strongly curved postero-prolateral cymbial apophysis (vs shorter and less curved); the elongated terminal apophysis with a groove and a lateral tooth-like apophysis (vs flattened terminal apophysis without the groove and the lateral tooth-like apophysis); and the long spine-like retrolateral tibial apophysis tapering distally (vs finger-like with a sharp pointed and slightly curved tip) (Figs 4A–G, 5 cf. Ding et al. 2024: 113, figs 1A, B, 2A–D). The female resembles *S.jinggangshanensis* Liu, Chen, Xu & Peng, 2017 in having the C-shaped atrial rim and ventrolaterally located copulatory openings, but differ by the epigynal hood lacking protrusions (vs distinctly protruding); the inverted triangular median septum being relatively broad (vs narrow); the more smoothly inwardly twisted copulatory ducts (vs strongly coiled); and the well sclerotized atrial rim on both sides (vs weak) (Fig. 6C, D cf. Liu et al. 2017: 292, figs 1A–D, 2A, B).

##### Description.

**Male** (holotype, Sal-409). Habitus as in Fig. 4A, B. Total length 3.77. Carapace 1.73 long, 1.37 wide. Eye sizes and interdistances: AME 0.41, ALE 0.26, PME 0.06, PLE 0.26, AME–AME 0.05, AME–ALE 0.07, PME–PME 1.08, PME–PLE 0.38, AME–PME 0.55, AME–PLE 0.98, ALE–ALE 0.90, PLE–PLE 1.20, ALE–PLE 0.77. MOA: 0.81 long; 0.90 anterior width, 1.14 posterior width. Chelicerae (Fig. 4B) with two promarginal teeth and one large triangular retromarginal tooth. Sternum (Fig. 4B) shield-shaped, longer than wide, anterior margin slightly straight. Leg measurements: I 5.92 (1.93, 1.93, 1.41, 0.32, 0.33); II 3.17 (1.02, 0.47, 0.69, 0.56, 0.43); III 2.79 (0.90, 0.42, 0.76, 0.41, 0.30); IV 4.13 (1.28, 0.55, 1.13, 0.90, 0.27). Femur width: I 0.46; II 0.24; III 0.18; IV 0.25. Leg spination (Fig. 4A–C): I tipv 1-2-1, rv 1-2-1; metpv 1-0-1, rv 1-0-1. Pedicel 0.08. Abdomen 1.96 long, 0.74 wide.

***Coloration*** (Fig. 4A, B). Carapace yellowish to yellowish-brown dorsally; eye bases surrounded by black pigment. Fovea distinctly arcuate, followed by several radial rows of punctate striae. Chelicerae pale yellow. Endites pale yellow, longer than wide, anterior margin arcuate. Labium as long as wide, semicircular, widely separated from sternum. Sternum yellow, with dark brown mottled spots around margin. Legs yellowish to yellowish-brown; leg I robust and swollen, with distinctly protruding coxa and trochanter. Abdomen relatively narrow, with distinct black markings dorsally; other markings inconspicuous. Spinnerets yellow.

***Palp*** (Figs 4D–G, 5). Ventral femoral apophysis tooth-like, with a short and pointed tip. Patella swollen, with a length–width ratio of ca 1.44. Retrolateral tibial apophysis spine-like, relatively long, nearly as long as half of cymbial length. Dorso-prolateral tibial apophysis broad in dorsal view, pointing toward the postero-prolateral cymbial apophysis. Postero-prolateral cymbial apophysis robust and slightly curved. Postero-retrolateral cymbial apophysis mastoid-like, short. Tegulum broad, with a clear mastoid-like apophysis in retrolateral view. Terminal apophysis boat-shaped with the slightly curved tip, the apical part densely covered with scale-like serrations and bearing barb-like projections sub-medio-laterally. Embolus broad at base, S-shaped, without spiral.

**Female** (paratype, Sal-401). Habitus as in Fig. 6A, B. As in male, except as noted. Total length 3.11. Carapace 1.55 long, 1.07 wide. Eye sizes and interdistances: AME 0.35, ALE 0.20, PME 0.07, PLE 0.20, AME–AME 0.08, AME–ALE 0.08, PME–PME 0.88, PME–PLE 0.28, AME–PME 0.46, AME–PLE 0.78; ALE–ALE 0.75, PLE–PLE 0.98, ALE–PLE 0.64. MOA: 0.68 long; 0.73 anterior width; 0.98 posterior width. Sternum (Fig. 6B) shield-shaped, longer than wide, posterior end triangular. Leg measurements: I 3.44 (1.04, 0.82, 0.86, 0.46, 0.26); II 2.21 (0.66, 0.22, 0.62, 0.47, 0.24); III 2.60 (0.78, 0.32, 0.50, 0.64, 0.36); IV 3.59 (1.00, 0.41, 0.87, 0.86, 0.45). Pedicel 0.06. Femur width: I 0.32; II 0.21; III 0.21; IV 0.28. Leg spination: I tipv 1-2-1, rv 1-2-1; metpv 1-0-1, rv 1-0-1. Abdomen 1.50 long, 0.90 wide.

***Coloration*** (Fig. 6A, B). Abdomen with a broad white stripe medially.

***Epigyne*** (Fig. 6C, D). Epigynal plate mushroom-like, with relatively large atrium. Atrial rim bowl-like, distinctly sclerotized bilaterally. Epigynal hood short mastoid-shaped, arising from anteromedial atrial rim. Median septum triangular, slightly shorter than 1/2 length of epigynal plate. Copulatory openings located on the bilateral part of median septum. Copulatory ducts S-shaped, anterior part extending anteriorly, then gently descending before forming an inward C-shaped loop, the posterior part tube-shaped, slightly convergent. Spermathecae boat-like, slightly separated. Glandular appendages short rod-shaped, transversally extending. Fertilization ducts relatively long, nearly as long as 1/2 length of spermathecae, directed laterally.

##### Distribution.

Known only from the type locality in Jiangxi Province, China (Fig. 16).

##### Etymology.

The name is taken from the type locality, noun in apposition.

#### ﻿ Synagelides himalaicus

Taxon classificationAnimaliaAraneaeSalticidae

Bohdanowicz, 1987, stat. res.

9DB65B62-0C82-59D4-B564-E604B8DDB61A

Figs 7 , 8 , 9

Synagelides himalaicus Bohdanowicz, 1987: 76, figs 35–44 (male).
Synagelides jiricus Bohdanowicz, 1987: 83, figs 62–63 (female). Syn. nov.
Synagelides martensi Logunov & Hereward, 2006: 287, figs 33, 37–40.

##### Additional material examined.

• 1 ♂ (Sal-410, ASM-JGSU), 27°28'20.68"N, 88°54'29.28"E, 2947 m a.s.l., Chunpei Village, Xarsingma Town, Yadong County, Shigatse City, Tibet Autonomous Region, China, 4 August 2023, K. Liu, Z. Jiang & Y. Yao leg.; • 1 ♂ 3 ♀ (Sal-410, ASM-JGSU), same data as previous; • 1 ♂ 1 ♀ (Sal-411, ASM-JGSU), 27°29'33.38"N, 88°54'18.16"E, 2958 m a.s.l., near Yadong County Government, other data as previous.

##### Diagnosis.

**Figure 7.**
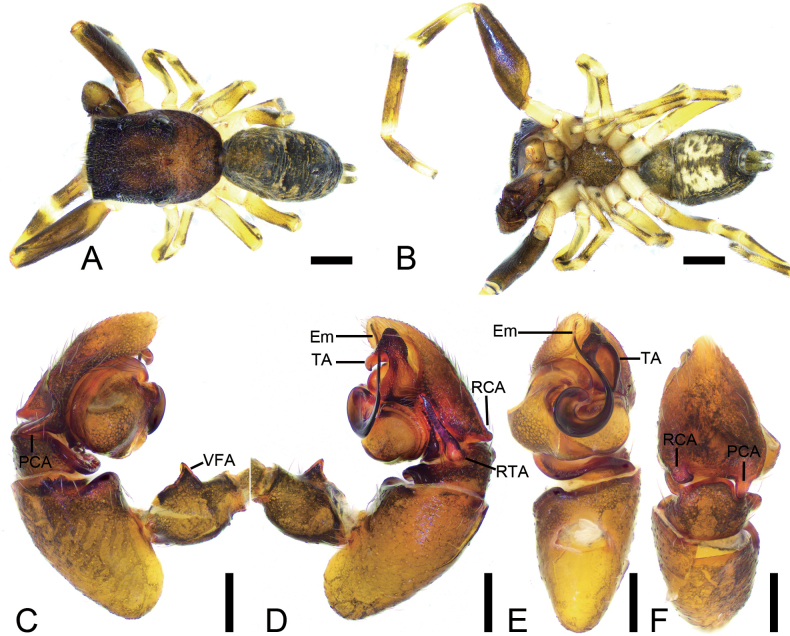
*Synagelideshimalaicus* Bohdanowicz, 1987, male. **A.** Habitus, dorsal view; **B.** Same, ventral view; **C.** Palp, prolateral view; **D.** Same, retrolateral view; **E.** Same, ventral view; **F.** Same, dorsal view. Abbreviations: Em – embolus, PCA – postero-prolateral cymbial apophysis, RCA – postero-retrolateral cymbial apophysis, RTA – retrolateral tibial apophysis, TA – terminal apophysis, VFA – ventral femoral apophysis. Scale bars: 0.5 mm (**A, B**); 0.2 mm (**C–F**).

**Figure 8.**
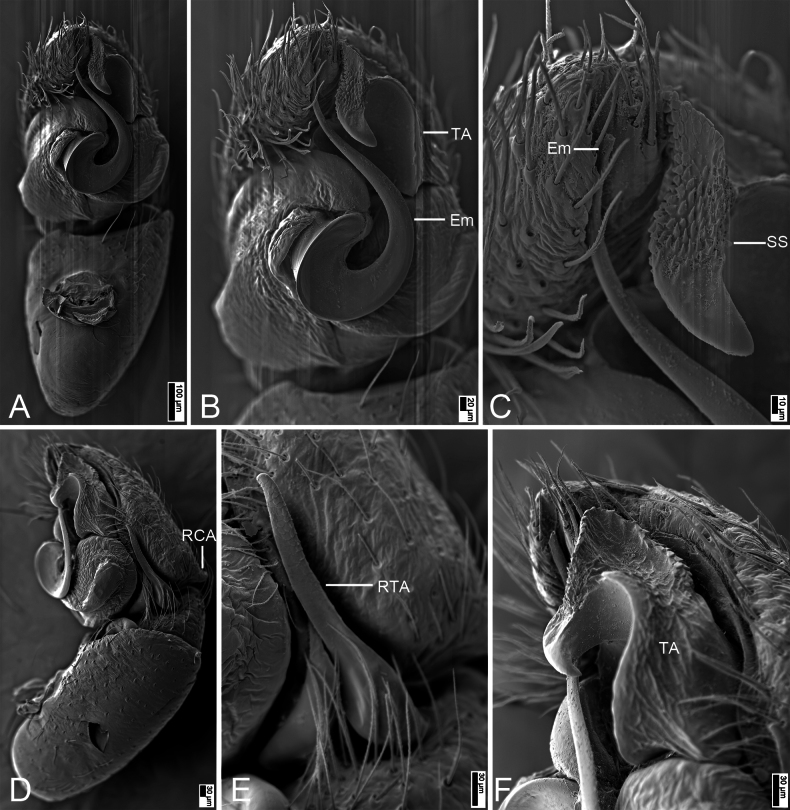
*Synagelideshimalaicus* Bohdanowicz, 1987, SEM images of the paratype male, left palp. **A.** Palp, ventral view; **B.** Same, detail of terminal apophysis and embolus; **C.** Same, detail of terminal apophysis and embolus; **D.** Same, retrolateral view; **E.** Same, detail of retrolateral tibial apophysis; **F.** Same, detail of terminal apophysis and embolus. Abbreviations: Em – embolus, RCA – postero-retrolateral cymbial apophysis, RTA – retrolateral tibial apophysis, SS – scale-like serrations, TA – terminal apophysis. Scale bars: 100 μm (**A**); 20 μm (**B**); 10 μm (**C**); 30 μm (**D–F**).

**Figure 9.**
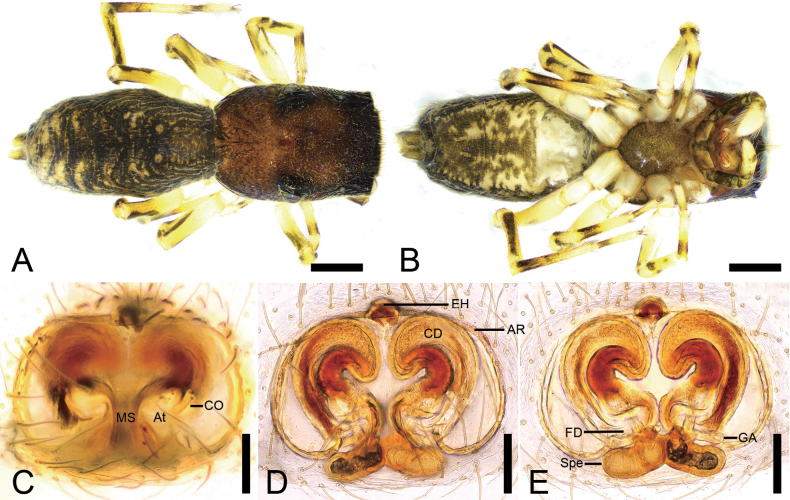
*Synagelideshimalaicus* Bohdanowicz, 1987, female. **A.** Habitus, dorsal view; **B.** Same, ventral view; **C.** Epigyne, ventral view; **D.** Same, ventral view; **E.** Same, dorsal view. Abbreviations: AR – atrial rim, At – atrium, CD – copulatory duct, CO – copulatory opening, EH – epigynal hood, FD – fertilization duct, GA – glandular appendage, MS – median septum, Spe – spermatheca. Scale bars: 0.5 mm (**A, B**); 0.1 mm (**C–E**).

The male resembles that of *Synagelidesmartensi* Bohdanowicz, 1987 in having flattened discoid embolus and hook-shaped terminal apophysis, but can be distinguished by the terminal apophysis blunt apically and densely covered with scale-like serrations (vs sharp and smooth); the robust postero-prolateral cymbial apophysis with a blunt apex (vs slender with a sharply pointed tip); and the retrolateral tibial apophysis slightly curved ventroapically (vs strongly curved ventrally and extending) (Figs 7C–F, 8 cf. Logunov and Hereward 2006: 287, figs 33, 37–40). The female of this species is similar to that of *S.martensi* Bohdanowicz, 1987 in having the apple-shaped epigynal plate, but differs in the ovoid atrium (vs crescent-shaped); the very wide atrial rim (vs moderately wide); the broad median septum that is inverted T-shaped (vs narrow and bottleneck-shaped); and the robust copulatory ducts with posterior part curved medially (vs slender and posterior part vertical) (Fig. 9C–E cf. Bohdanowicz 1987: 68, figs 8, 9).

##### Description.

**Male** (Sal-410). Habitus as in Fig. 7A, B. Total length 3.18. Carapace 1.65 long, 1.18 wide. Eye sizes and interdistances: AME 0.27, ALE 0.17, PME 0.06, PLE 0.19, AME–AME 0.10, AME–ALE 0.08, PME–PME 0.82, PME–PLE 0.40, AME–PME 0.43, AME–PLE 0.90, ALE–ALE 0.68, PLE–PLE 0.96, ALE–PLE 0.75. MOA: 0.60 long; 0.63 anterior width, 0.89 posterior width. Chelicerae (Fig. 7B) with two promarginal teeth and one large retromarginal tooth. Sternum (Fig. 7B) oval, longer than wide, anterior margin nearly arcuate. Leg measurements: I 4.63 (1.45, 1.21, 1.11, 0.50, 0.36); II 2.34 (0.73, 0.41, 0.47, 0.47, 0.26); III 2.63 (0.78, 0.41, 0.53, 0.63, 0.28); IV 3.64 (0.97, 0.55, 0.87, 0.86, 0.39). Femur width: I 0.46; II 0.25; III 0.19; IV 0.27. Leg spination: I ti pv1-3-1, rv1-3-1; met pv1-0-1, rv1-0-1. Pedicel 0.06. Abdomen 1.48 long, 0.94 wide.

***Coloration*** (Fig. 7A, B). Carapace reddish-brown to dark brown dorsally. Eye bases surrounded by black pigmentation. Fovea distinct, red-brown, followed by several radial rows of punctate striae. Chelicerae yellowish-brown, densely covered with yellow punctate spots. Endites yellowish-brown. Labium yellow. Sternum dark brown, mottled, with densely dark brown mottled spots. Legs yellowish-brown to reddish-brown, each segment with dark brown stripe, femur I robust and swollen, with abundant colorful setae. Abdomen dorsally with one pair of distinct yellow sigilla and several yellow brown markings; venter with a dark brown median stripe. Spinnerets yellow-brown, mottled.

***Palp*** (Figs 7C–F, 8). Femur with a strong tooth-like ventral apophysis. Patella swollen, with a length–width ratio of ca 2.75. Retrolateral tibial apophysis finger-like, with a sharp pointed tip, nearly as long as 1/2 length of cambium. Postero-retrolateral cymbial apophysis finger-shaped in dorsal view, straight, apex directed at the groove of tibia. Postero-prolateral cymbial apophysis broad, hook-shaped in dorsal view. Tegulum broad, with two extensions at the base of embolus in ventral view. Terminal apophysis arising from antero-retrolateral part of tegulum, strongly sclerotized, hook-shaped, and bearing distinct scale-like serrations apically. Embolus with a flattened and spiraling base, gradually tapering distally, its slender apex slightly curved retrolaterally to terminal apophysis.

**Female** (Sal-410). Habitus as in Fig. 9A, B. As in male, except as noted. Total length 3.45. Carapace 1.60 long, 1.16 wide. Eye sizes and interdistances: AME 0.29, ALE 0.18, PME 0.05, PLE 0.22, AME–AME 0.07, AME–ALE 0.09, PME–PME 0.87, PME–PLE 0.41, AME–PME 0.36, AME–PLE 0.81, ALE–ALE 0.70, PLE–PLE 0.99, ALE–PLE 0.69. MOA: 0.58 long; 0.62 anterior width, 0.93 posterior width. Sternum (Fig. 9B) shield-shaped, longer than wide, anterior margin arcuate. Leg measurements: I 3.58 (1.11, 0.84, 0.87, 0.47, 0.29); II 2.55 (0.79, 0.42, 0.51, 0.54, 0.29); III 2.67 (0.79, 0.37, 0.57, 0.59, 0.35); IV 4.00 (1.31, 0.51, 0.92, 0.83, 0.43). Femur width: I 0.40; II 0.25; III 0.25; IV 0.30. Leg spination: I ti pv1-2-1, rv1-2-1; met pv1-0-1, rv1-0-1. Pedicel 0.06. Abdomen 1.88 long, 1.09 wide.

***Coloration*** (Fig. 9A, B). Abdomen dorsally with four chevron-shaped stripes; venter with several white markings.

***Epigyne*** (Fig. 9C–E). Epigynal plate apple-shaped, with an ovoid atrium. Atrial rim ear-like, very wide. Epigynal hood short arc-shaped, arising from anteromedial atrial rim. Median septum inverted T-shaped, posterior part slightly broadened. Copulatory openings subtriangular, located at anterolateral part of median septum. Copulatory ducts S-shaped, anterior and medial part with equal width, posterior part C-shaped, anteriorly swollen, touching each other. Glandular appendages long and broad, longer than 2/3 length of spermathecae. Spermathecae large, elongated, swollen, inner margin close touching forming a triangle. Fertilization ducts relatively long, longer than 1/2 length of spermathecae, directed laterally.

##### Distribution.

Known from the type locality in Nepal (Bohdanowicz 1987), and Tibet Autonomous Region, China (Fig. 16).

##### Remarks.

It should be noted that the male of *S.himalaicus* Bohdanowicz, 1987 illustrated by Bohdanowicz (1987) is identical to our material collected from Yadong County, Tibet Autonomous Region. However, our female specimens are similar to illustrations provided in the same work for *S.jiricus* Bohdanowicz, 1987. Both of these names were considered synonyms of *S.martensi* Bohdanowicz, 1987 by Logunov and Hereward (2006), which is rejected here (see “Diagnosis”). Consequently, *S.himalaicus* Bohdanowicz, 1987 is reinstated as a valid species, with *S.jiricus* syn. nov. proposed as its synonym. This represents the first record of this species in China.

#### ﻿ Synagelides kangding

Taxon classificationAnimaliaAraneaeSalticidae

K. K. Liu sp. nov.

736BC7AF-4EC8-55F7-BC72-5D5386F1C6B2

https://zoobank.org/5343DA3D-F4EA-46F7-9121-615DB2D70813

Figs 10 , 11 , 12

##### Material examined.

***Holotype*** • 1 ♂ (Sal-412, ASM-JGSU), 30°03'53.44"N, 102°00'16.62"E, 2399 m a.s.l., Caiyuanzi Village, National Highway G318, Kangding City, Ganzi Tibetan Autonomous Prefecture, Sichuan Province, China, 26 July 2023, K. Liu, Z. Jiang & Y. Yao leg. ***Paratypes*** • 3 ♂ 1 ♀ (Sal-412, ASM-JGSU), same data as holotype.

##### Diagnosis.

**Figure 10.**
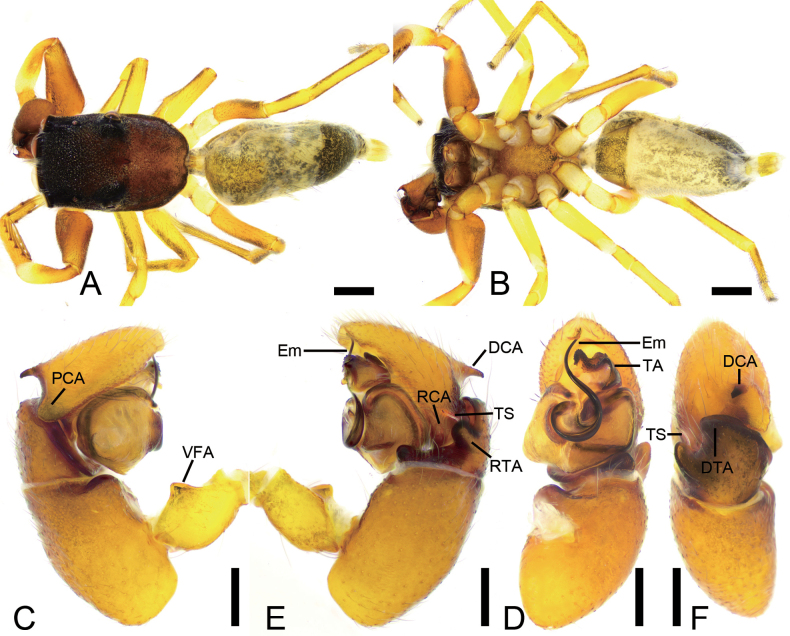
*Synagelideskangding* sp. nov., male holotype. **A.** Habitus, dorsal view; **B.** Same, ventral view; **C.** Palp, prolateral view; **D.** Same, ventral view; **E.** Same, retrolateral view; **F.** Same, dorsal view. Abbreviations: DCA – dorsal cymbial apophysis, DTA – dorso-prolateral tibial apophysis, Em – embolus, PCA – postero-prolateral cymbial apophysis, RCA – postero-retrolateral cymbial apophysis, RTA – retrolateral tibial apophysis, TA – terminal apophysis, TS – tibial spine, VFA – ventral femoral apophysis. Scale bars: 0.5 mm (**A, B**); 0.2 mm (**C–F**).

**Figure 11.**
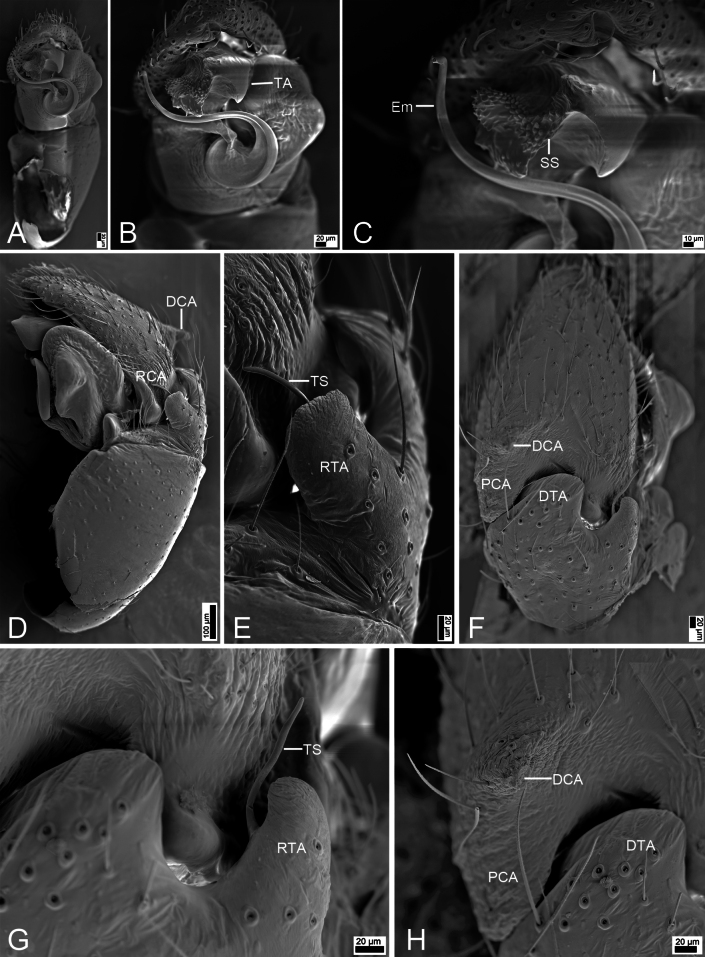
*Synagelideskangding* sp. nov., SEM images of the paratype male palps. **A.** Left palp, ventral view; **B.** Same, detail of terminal apophysis and embolus; **C.** Same, detail of terminal apophysis and embolus; **D.** Same, retrolateral view; **E.** Same, detail of retrolateral tibial apophysis and tibial spine; **F.** Right palp, detail of dorso-prolateral tibial apophysis and postero-prolateral cymbial apophysis; **G.** Same, detail of retrolateral tibial apophysis and tibial spine; **H.** Same, detail of dorso-prolateral tibial apophysis and postero-prolateral cymbial apophysis. Abbreviations: DCA – dorsal cymbial apophysis, DTA – dorso-prolateral tibial apophysis, Em – embolus, PCA – postero-prolateral cymbial apophysis, RCA – postero-retrolateral cymbial apophysis, RTA – retrolateral tibial apophysis, SS – scale-like serrations, TS – tibial spine, TA – terminal apophysis. Scale bars: 30 μm (**A**); 20 μm (**B, E–H**); 10 μm(**C**); 100 μm (**D**).

**Figure 12.**
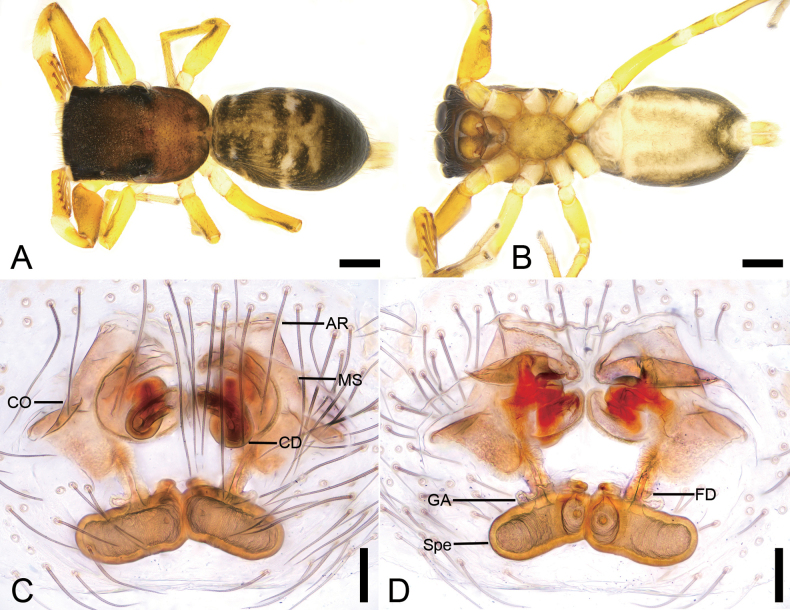
*Synagelideskangding* sp. nov., female paratype. **A.** Habitus, dorsal view; **B.** Same, ventral view; **C.** Epigyne, ventral view; **D.** Same, dorsal view. Abbreviations: AR – atrial rim, CD – copulatory duct, CO – copulatory opening, FD – fertilization duct, GA – glandular appendage, MS – median septum, Spe – spermatheca. Scale bars: 0.5 mm (**A, B**); 0.2 mm (**C, D**).

The male of this new specie resembles that of *Synagelideszhilcovae* Prószyński, 1979 in having flattened discoid embolus, but can be distinguished by the abdominal dorsum exhibiting yellowish-brown anterior two-thirds and dark brown posterior third (vs entirely black with three white chevron stripes); dorsal cymbial apophysis sharply spine-like and short (vs lacking); the terminal apophysis bearing scale-like serrations apically (vs smooth); and the retrolateral tibial apophysis bearing a tibial spine positioned medially (vs only tibial spine present) (Figs 10A–F, 11 cf. Omelko and Fomichev 2021: 99, figs 6–10, 13, 14, 18–20, 25–28). It also resembles *S.hubeiensis* Peng & Li, 2008 in having sharply spine-like dorsal cymbial apophysis, but can be distinguished by the terminal apophysis triangular (vs C-shaped); and the retrolateral tibial apophysis bearing a tibial spine positioned medially (vs lacking) (Figs 10C–F, 11 cf. Peng, 2020: 450, fig. 328a–e). The female of this new specie is similar to that of *S.subagoriformis* Li, Wang & Peng, 2021 in having the trapezoidal median septum, but differs in the epigynal plate hexagonal (vs subtrapezoidal); the median septum wide (vs narrow); the copulatory ducts medial part membranous (vs sclerotized); and glandular appendages long, approximately as long as 2/3 length of spermathecae(vs short and as long as 1/2 length of spermathecae) (Fig. 12C, D cf. Li et al. 2021: 182, fig. 5A–C, F).

##### Description.

**Male** (holotype, Sal-412). Habitus as in Fig. 10A, B. Total length 4.16. Carapace 1.89 long, 1.26 wide. Eye sizes and interdistances: AME 0.38, ALE 0.22, PME 0.07, PLE 0.23, AME–AME 0.08, AME–ALE 0.04, PME–PME 0.92, PME–PLE 0.35, AME–PME 0.60, AME–PLE 0.95, ALE–ALE 0.81, PLE–PLE 1.07, ALE–PLE 0.76. MOA: 0.77 long; 0.79 anterior width, 1.01 posterior width. Chelicerae (Fig. 10B) with two promarginal teeth and one large triangular retromarginal tooth. Sternum (Fig. 10B) oval-shaped, longer than wide. Leg measurements: I 4.63 (1.38, 1.16, 1.17, 0.56, 0.36); II 3.09 (0.99, 0.46, 0.68, 0.53, 0.43); III 3.25 (0.98, 0.46, 0.70, 0.74, 0.37); IV 4.55 (1.34, 0.61, 1.13, 1.05, 0.42). Femur width: I 0.52; II 0.26; III 0.25; IV 0.35. Leg spination: I ti pv1-3-1, rv1-3-1; met pv1-0-1, rv1-0-1. Pedicel 0.21. Abdomen 2.08 long, 1.02 wide.

***Coloration*** (Fig. 10A, B). Carapace reddish-brown to dark brown dorsally, broader anteriorly. Eye bases surrounded by black pigmentation. Fovea distinct, followed by several radial rows of punctate striae. Chelicerae yellowish-brown. Endites yellowish-brown, length approximately equal to width. Labium yellow-brown. Sternum yellowish-brown, densely covered with black punctate spots. Legs yellowish-brown to reddish-brown; Leg I reddish-brown with robust and swollen femur. Abdomen dorsally with yellowish-brown anterior 2/3 and dark brown posterior third; venter pale yellow with dark brown anterior region, densely covered with black punctate spots. Spinnerets yellow, mottled.

***Palp*** (Figs 10C–F, 11). Femur with a relatively small blunt, tooth-like ventral apophysis, slightly shorter than femoral width. Patella swollen, with a length–width ratio of ca 1.69. Retrolateral tibial apophysis digitiform, with a blunt tip and a tibial spine located medially. Dorso-prolateral tibial apophysis very broad in dorsal view, pointing toward the retrolateral tibial apophysis. Postero-retrolateral cymbial apophysis tooth-like, pointing toward the retrolateral tibial apophysis. Postero-prolateral cymbial apophysis digitiform and relatively broad. Dorsal cymbial apophysis sharply spine-like and short. Tegulum broad, prolaterally and retrolaterally protruded. Terminal apophysis with a groove, arising from antero-retrolateral part of tegulum, strongly sclerotized, triangular, bearing scale-like serrations apically. Embolus slender, with a flattened discoid base, curved medially in its mid-proximal portion, gradually tapering distally, and terminating in an apical half-coil around the margin of the terminal apophysis.

**Female** (paratype, Sal-412). Habitus as in Fig. 12A, B. As in male, except as noted. Total length 3.97. Carapace 1.81 long, 1.24 wide. Eye sizes and interdistances: AME 0.32, ALE 0.22, PME 0.07, PLE 0.22, AME–AME 0.11, AME–ALE 0.07, PME–PME 1.00, PME–PLE 0.33, AME–PME 0.43, AME–PLE 0.82, ALE–ALE 0.78, PLE–PLE 1.08, ALE–PLE 0.71. MOA: 0.66 long; 0.72 anterior width, 1.10 posterior width. Sternum (Fig. 12B) oval-shaped, longer than wide, anterior margin subarcuate. Leg measurements: I 3.88 (1.22, 0.83, 0.95, 0.47, 0.41); II 2.86 (0.85, 0.48, 0.63, 0.55, 0.35); III 2.91 (0.91, 0.44, 0.69, 0.52, 0.35); IV 4.36 (1.21, 0.60, 1.06, 0.98, 0.51). Femur width: I 0.41; II 0.24; III 0.25; IV 0.30. Leg spination: I ti pv1-3-1, rv1-3-1; met pv1-0-1, rv1-0-1. Pedicel 0.03. Abdomen 2.13 long, 1.29 wide.

***Coloration*** (Fig. 12A, B). Abdomen yellowish-brown to dark brown, dorsally with two pairs of yellowish longitudinal stripes laterally along the midline; venter smooth, pale yellow, with two broad longitudinal brown bands merged together caudally.

***Epigyne*** (Fig. 12C, D). Epigynal plate hexagonal. Median septum broad, trapezoidal. Atrial rim located along front margin of epigyne, slightly straight. Copulatory openings slit-like, located anterolaterally. Copulatory ducts, anterior part membranous and narrow, medial part membranous, broad and flattened, posterior part tube-shaped, shorter than spermathecae. Glandular appendages long, near the posterior copulatory ducts, approximately as long as 2/3 length of spermathecae. Spermathecae elongated oval, closely touching each other. Fertilization ducts relatively short, nearly as long as 1/2 length of spermathecae, directed laterally.

##### Distribution.

Known only from the type locality in Sichuan Province, China (Fig. 16).

##### Etymology.

The name is taken from the type locality, noun in apposition.

#### ﻿ Synagelides wuyuan

Taxon classificationAnimaliaAraneaeSalticidae

K. K. Liu sp. nov.

C5961773-1357-5930-9277-35AC5ADBB910

https://zoobank.org/08E30534-2CFA-43CD-B587-BC4E2E344253

Figs 13 , 14 , 15

##### Material examined.

***Holotype*** • 1 ♂ (Sal-413, ASM-JGSU), 29°31'36.50"N, 117°44'23.77"E, 720 m a.s.l., Konghou Bridge, Dazhangshan Township, Wuyuan Forest Birds National Nature Reserve, Wuyuan County, Shangrao City, Jiangxi Province, China, 2 March 2025, Z. Jiang, B. Liu & Z. Wang leg. ***Paratypes*** • 1 ♂ 3 ♀ (Sal-413, ASM-JGSU), same data as holotype; • 1 ♂ (Sal-413, ASM-JGSU), 29°32'18.24"N, 117°44'43.11"E, 909 m a.s.l., Zhangshan Village, 2 March 2025, other data same as previous.

##### Diagnosis.

**Figure 13.**
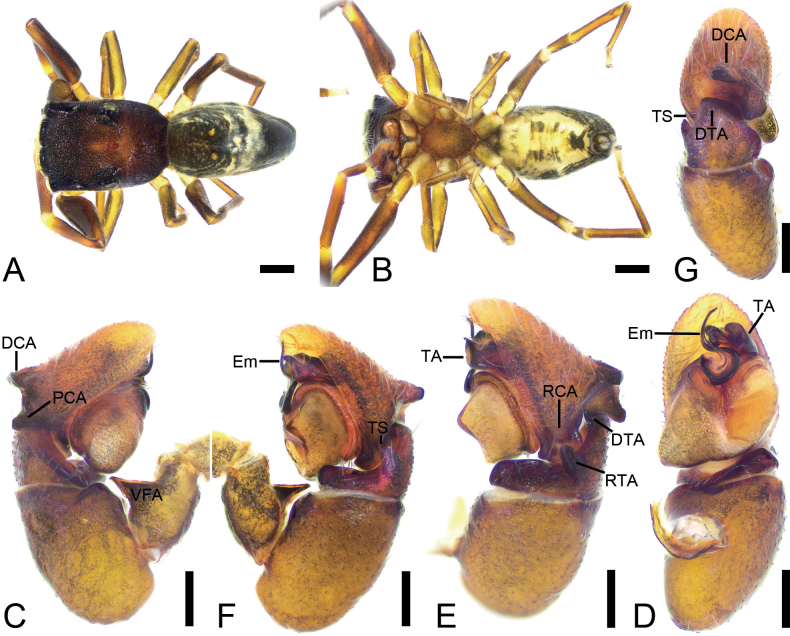
*Synagelideswuyuan* sp. nov., male holotype. **A.** Habitus, dorsal view; **B.** Same, ventral view; **C.** Palp, prolateral view; **D.** Same, ventral view; **E.** Same, retrolateral view, slightly dorsal; **F.** Same, retrolateral view; **G.** Same, dorsal view. Abbreviations: DCA – dorsal cymbial apophysis, DTA – dorso-prolateral tibial apophysis, Em – embolus, PCA – postero-prolateral cymbial apophysis, RCA – postero-retrolateral cymbial apophysis, RTA – retrolateral tibial apophysis, TA – terminal apophysis, TS – tibial spine, VFA – ventral femoral apophysis. Scale bars: 0.5 mm (**A, B**); 0.2 mm (**C–G**).

**Figure 14.**
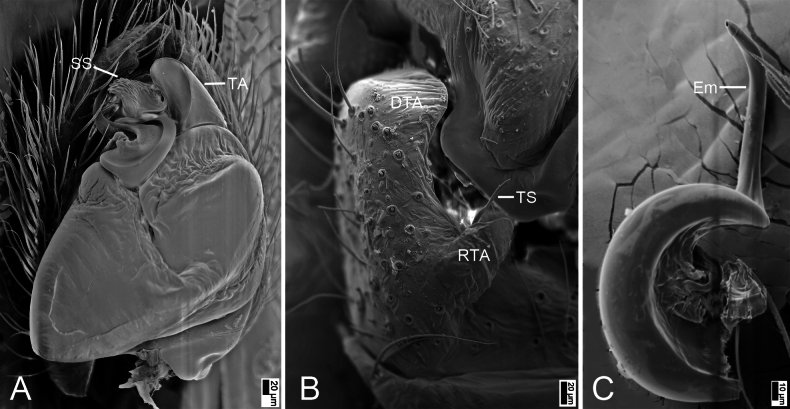
*Synagelideswuyuan* sp. nov., SEM images of the paratype male palps. **A.** Left palp, detail of terminal apophysis; **B.** Right palp, detail of retrolateral tibial apophysis and tibial spine; **C.** Same, detail of embolus. Abbreviations: DTA – dorso-prolateral tibial apophysis, Em – embolus, RTA – retrolateral tibial apophysis, SS – scale-like serrations, TS – tibial spine, TA – terminal apophysis. Scale bars: 20 μm (**A, B**); 10 μm (**C**).

**Figure 15.**
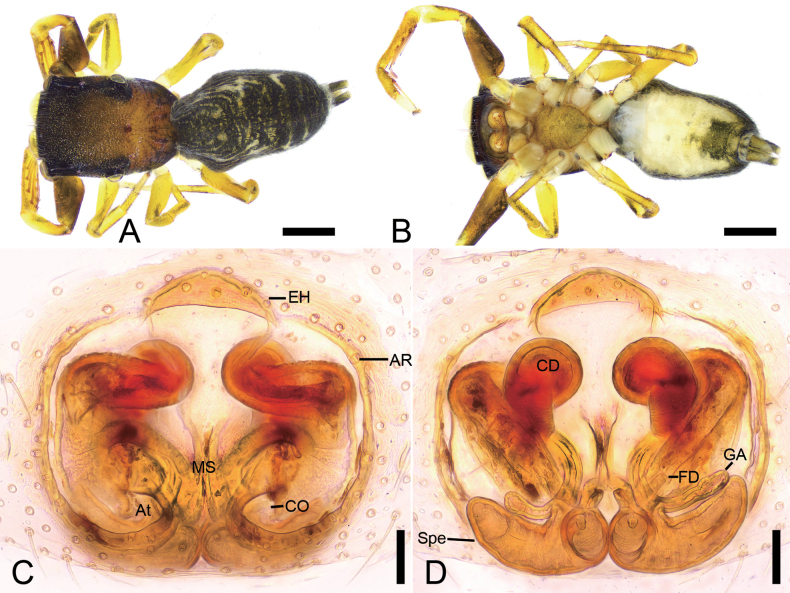
*Synagelideswuyuan* sp. nov., female paratype. **A.** Habitus, dorsal view; **B.** Same, ventral view; **C.** Epigyne, ventral view; **D.** Same, dorsal view. Abbreviations: AR – atrial rim, At – atrium, CD – copulatory duct, CO – copulatory opening, EH – epigynal hood, FD – fertilization duct, GA – glandular appendage, MS – median septum, Spe – spermatheca. Scale bars: 0.5 mm (**A, B**), 0.2 mm (**C, D**).

The male of this species is similar to that of *Synagelidessubagoriformis* Li, Wang & Peng, 2021 in having flattened discoid embolus and digitiform retrolateral tibial apophysis, but can be distinguished by the abdomen bearing two distinct white stripes (vs one); the postero-prolateral cymbial apophysis very large, horn-like (vs relatively small and pointed); the dorsal cymbial apophysis finger-like and blunt (vs subtriangular and sharp); and the retrolateral tibial apophysis bearing a tibial spine positioned medially (vs lacking) with considerable separation from the dorso-prolateral tibial apophysis (vs closely approximated) (Figs 13A–G, 14 cf. Li et al. 2021: 182, figs 4A–D, 5D, E). The female resembles *S.serratus* Liu, 2022 in having a broadly bell-shaped epigynal hood and the ear-shaped atrial rim, but differ by the black body coloration (vs pale yellow); the narrower epigynal atrium (vs broader), the anteriorly projecting copulatory ducts (vs normally curved); spermathecae dagger-shaped (vs oval and short glandular appendages); and glandular appendages very long, longer than 3/4 length of spermathecae (vs nearly 1/2 length of spermathecae) (Fig. 15A–D cf. Liu et al. 2022: 60, fig. 8A–D).

##### Description.

**Male** (holotype, Sal-413). Habitus as in Fig. 13A, B. Total length 3.03. Carapace 1.56 long, 1.09 wide. Eye sizes and interdistances: AME 0.31, ALE 0.20, PME 0.07, PLE 0.21, AME–AME 0.07, AME–ALE 0.06, PME–PME 0.89, PME–PLE 0.27, AME–PME 0.40, AME–PLE 0.72, ALE–ALE 0.71, PLE–PLE 0.92, ALE–PLE 0.59. MOA length 0.63, anterior width 0.67, posterior width 0.97. Chelicerae (Fig. 13B) with two promarginal teeth (proximal larger) and one bifurcated plate-shaped retromarginal tooth. Sternum (Fig. 13B) oval-shaped, longer than wide, anterior margin nearly arcuate. Leg measurements: I 3.15 (0.98, 0.70, 0.77, 0.41, 0.29), II 2.24 (0.64, 0.38, 0.45, 0.45, 0.32), III 2.31 (0.69, 0.30, 0.46, 0.58, 0.28), IV 3.16 (0.91, 0.45, 0.70, 0.78, 0.32). Femur width: I 0.36; II 0.22; III 0.21; IV 0.28. Leg spination: I tipv 1-3-1, rv 1-3-1; metpv 1-0-1, rv 1-0-1. Pedicel 0.05. Abdomen 1.65 long, 1.08 wide.

***Coloration*** (Fig. 13A, B). Carapace reddish-brown to dark brown dorsally. Eye bases surrounded by black pigment. Fovea distinct, followed by several radial rows of punctate striae. Chelicerae reddish-brown. Endites yellow to yellow brown. Labium yellowish to dark brown. Sternum reddish-brown to dark brown, mottled. Legs yellow to dark-brown to reddish-brown. Abdomen dorsally with a pair of distinct yellow sigilla in anterior half and two broad white transverse stripes medially; venter with a V-shaped dark brown stripe. Spinnerets dark brown, mottled.

***Palp*** (Figs 13C–G, 14). Femur with a strongly sharp tooth-like ventral apophysis, longer than femoral width. Patella swollen, with a length–width ratio of ca 1.63. Retrolateral tibial apophysis digitiform, blunt, with a tibial spine positioned medially. Dorso-retrolateral tibial apophysis horn-like with broad base, pointing at the base of postero-retrolateral cymbial apophysis. Postero-retrolateral cymbial apophysis well-developed and prominent, very broad, directed at the tibial groove. Postero-prolateral cymbial apophysis very large, trifurcate, dorsal one finger-like, directed retrolaterally, the others larger than dorsal. Tegulum broad, with a clear mastoid apophysis directed prolaterally in ventral view. Terminal apophysis strongly sclerotized, V-shaped in ventral view, arising from antero-retrolateral part of tegulum, with little scale-like serrations on anterior surface. Embolus with flattened discoid base, gradually tapering and curving medio-distally, medial part screw-like, sub-apical part turn around margin of terminal apophysis.

**Female** (paratype, Sal-413). Habitus as in Fig. 15A, B. As in male, except as noted. Total length 2.78. Carapace 1.38 long, 1.01 wide. Eye sizes and interdistances: AME 0.31, ALE 0.18, PME 0.05, PLE 0.17, AME–AME 0.03, AME–ALE 0.06, PME–PME 0.81, PME–PLE 0.25, AME–PME 0.41, AME–PLE 0.71, ALE–ALE 0.67, PLE–PLE 0.89, ALE–PLE 0.57. MOA length 0.6, anterior width 0.64, posterior width 0.9. Sternum (Fig. 15B) shield-shaped, longer than wide, posterior end triangular. Leg measurements: I 3.10 (0.87, 0.53, 0.79, 0.66, 0.25); II 2.02 (0.63, 0.35, 0.42, 0.40, 0.22); III 2.16 (0.65, 0.30, 0.40, 0.53, 0.28); IV 2.24 (0.65, 0.32, 0.45, 0.53, 0.29). Femur width: I 0.34; II 0.18; III 0.19; IV 0.24. Leg spination: I: tipv 1-2-1, rv 1-2-1; metpv 1-0-1, rv 1-0-1. Pedicel 0.08. Abdomen 1.46 long, 0.94 wide.

***Coloration*** (Fig. 15A, B). Darker than male. Dorsum of abdomen with several faint white stripes; venter smooth, pale yellow.

***Epigyne*** (Fig. 15C, D). The epigynal plate jar-like, with a broadly bell-shaped epigynal hood. Atrial rim ear-shaped. Atrium ovoid, separated by the median septum. Median septum slit-like, relatively narrow. Copulatory openings posteromedially located, nearly horseshoe-shaped. Copulatory ducts relatively thick and intertwined, with the anterior portions extending from posteromedial to anterolateral part of valve, medial part spiral, posterior part thin and C-shaped. Spermathecae dagger-shaped, large, swollen, closely touching, directed anterolaterally. Glandular appendages very long, longer than 3/4 length of spermathecae. Fertilization ducts relatively broad, nearly as long as 1/3 length of spermathecae, directed anteriorly.

##### Distribution.

**Figure 16.**
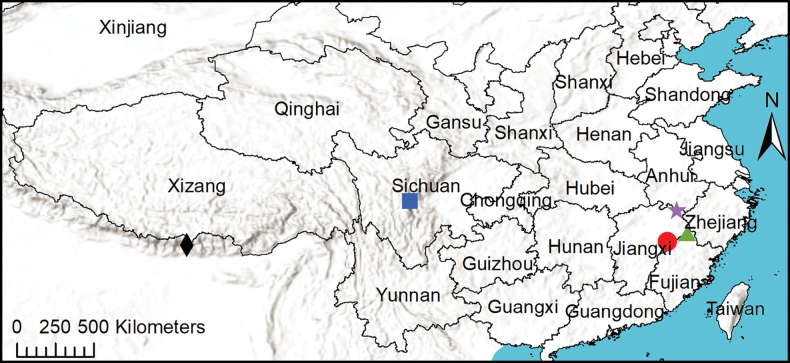
Collection sites of *Synagelidesdajueshan* sp. nov. (red circle), *S.guangfeng* sp. nov. (green triangle), *S.himalaicus* Bohdanowicz, 1987 (black rhombus), *S.kangding* sp. nov. (blue square), and *S.wuyuan* sp. nov. (purple star) in China.

Known only from the type locality in Jiangxi Province, China (Fig. 16).

##### Etymology.

The species name refers to the type locality; noun in apposition.

## ﻿Discussion

The genus *Synagelides*, a group of small-sized and highly habitat-specialized jumping spiders, currently comprises 90 described species including the five species described herein (WSC 2025; this study), with a distinctly concentrated distribution in East and Southeast Asia. Apart from a few species recorded in northern India, Nepal, and the Indochinese Peninsula, mainland China (including Taiwan, Hainan, and other islands) constitutes the modern distribution core of this genus (WSC 2025; The Biodiversity Committee of Chinese Academy of Sciences 2024). The frequency of new species discoveries in China has continued to rise in recent decades (Logunov and Hereward 2006; Logunov 2017).

Based on historical records and newly acquired data, the distribution of *Synagelides* in China (Li and Lin 2016 ; The Biodiversity Committee of Chinese Academy of Sciences 2024; WSC 2025) exhibits the following characteristics:

1. species diversity is concentrated in the mountainous regions of southwestern China (Yunnan, Guizhou, Sichuan, and Tibet) and the hilly areas along the middle-lower Yangtze River basin (Jiangxi, Hunan, Hubei). Yunnan Province stands out as a particularly rich region, with 15 recorded species (The Biodiversity Committee of Chinese Academy of Sciences 2024), likely due to its complex topography and vertical climatic gradients that facilitate niche differentiation;
2. the newly reported species from Tibet (*S.himalaicus*) and Sichuan (*S.kangding* sp. nov.) fill distribution gaps in the southeastern margin of the Qinghai-Tibet Plateau and the Hengduan Mountains. This suggests that the genus may have expanded its geographic range by adapting to high-elevation humid habitats, such as forest-edge shrubs and moss layers;
3. isolated populations on Taiwan and Hainan (e.g. *S.hainanensis*) (Peng 2020; Wang et al. 2020) imply that geographic isolation may drive speciation on islands, though molecular phylogenetic evidence is required to confirm this hypothesis.

The three new species from Jiangxi, *S.dajueshan* sp. nov., *S.guangfeng* sp. nov. and *S.wuyuan* sp. nov., sympatrically distributed in the mountainous regions of southern-central Jiangxi, further expand the diversity distribution of the genus within the hilly landscapes of this province. The discovery of *S.himalaicus* in Tibet holds particular biogeographic significance: its habitat at nearly or more than 3,000 meters above sea level (Logunov and Hereward 2006; this study) exceeds the previously recognized altitudinal limit for *Synagelides* (typically < 2,500 m), suggesting unique physiological adaptations, such as cold tolerance or shortened life cycles, enabling survival in extreme high-elevation environments.

## Supplementary Material

XML Treatment for Synagelides dajueshan

XML Treatment for Synagelides guangfeng

XML Treatment for Synagelides himalaicus

XML Treatment for Synagelides kangding

XML Treatment for Synagelides wuyuan

## ﻿Additional information

### Use of AI

No use of AI was reported.

### Author ORCIDs

Bin-Lu Liu https://orcid.org/0009-0009-3080-8987

Xiao-Qi Ning https://orcid.org/0009-0006-3116-8529

Shi-Hui Huang https://orcid.org/0009-0000-5334-3638

Yan-Bin Yao https://orcid.org/0000-0002-2560-9299

Chang-Yong Lin https://orcid.org/0009-0001-1055-6518

Jun Yang https://orcid.org/0009-0007-6540-2229

Ke-Ke Liu https://orcid.org/0000-0001-7822-3667

### Data availability

All of the data that support the findings of this study are available in the main text.
